# Early Childhood Caries and Oral Health-Related Quality of Life in Preschool Children: A Systematic Review

**DOI:** 10.3390/jcm15114314

**Published:** 2026-06-02

**Authors:** Paula Piekoszewska-Ziętek, Anna Turska-Szybka, Aleksandra Szczepanik, Dorota Olczak-Kowalczyk

**Affiliations:** 1 Department of Pediatric Dentistry, Medical University of Warsaw, 02-097 Warsaw, Poland; ppiekoszewska@wum.edu.pl (P.P.-Z.); dorota.olczak-kowalczyk@wum.edu.pl (D.O.-K.); 2 Students’ Research Group, Department of Pediatric Dentistry, Medical University of Warsaw, 02-097 Warsaw, Poland; pedodoncja@wum.edu.pl

**Keywords:** OHRQoL, ECOHIS, children, ECC

## Abstract

**Objectives**: To systematically review the evidence on the association between early childhood caries (ECC) and oral health-related quality of life (OHRQoL) in preschool children, focusing on studies using the Early Childhood Oral Health Impact Scale (ECOHIS) or its validated language versions. **Methods**: This systematic review was conducted and reported in accordance with the PRISMA 2020 guidelines. Electronic searches identified primary observational studies published within the last 10 years. Eligible studies included preschool children, clinically assessed ECC/caries, and OHRQoL measured with ECOHIS or a validated, adapted version. Data extraction covered study characteristics, caries assessment, OHRQoL measures, and main findings. Risk of bias was assessed using the Joanna Briggs Institute appraisal tools. **Results**: Twenty studies were included (19 cross-sectional and 1 cohort) with sample sizes ranging from 151 to 1783 participants. Across diverse populations and settings, ECC was consistently associated with poorer OHRQoL in preschool children and their families. Greater caries severity, untreated lesions, and advanced disease consequences were generally linked to higher ECOHIS scores. The most affected domains were pain, eating and drinking difficulties, sleep disturbance, irritability, and family distress. **Conclusions**: The available evidence consistently indicates that ECC is associated with poorer OHRQoL in preschool children and their families. From a clinical perspective, incorporating OHRQoL measures into pediatric dental assessment may improve recognition of disease burden beyond clinical indices alone.

## 1. Introduction

Early childhood caries (ECCs) remains one of the most prevalent and avoidable oral diseases in children younger than 6 years of age. It represents a major public health problem worldwide, with dental caries affecting an estimated 510 million children in the primary dentition, and its burden continues to be shaped by biological, behavioral, psychosocial and socioeconomic factors [1,2].

Beyond its clinical presentation, ECC may negatively affect a child’s daily functioning and family well-being. Untreated carious lesions may lead to pain, eating difficulties, sleep disturbances, irritability and impaired quality of life, while caregivers may report distress, guilt, disrupted work routines and reduced family functioning [3,4,5]. Recent studies in preschool populations have consistently shown that greater caries experience and more severe untreated lesions are associated with worse child- and family-level outcomes [6,7].

Oral health-related quality of life (OHRQoL) has become an important patient-centered outcome in pediatric dentistry. In preschool children, OHRQoL is usually assessed using proxy-reported measures completed by parents or caregivers, because children at this age may have limited ability to reliably report the impact of oral conditions on everyday life. The Early Childhood Oral Health Impact Scale (ECOHIS) was specifically developed to assess the impact of oral health problems on the quality of life of preschool children and their families and remains one of the most widely used instruments for this purpose [8,9].

Although the association between dental caries and OHRQoL has been widely explored, the available literature includes different age groups, diverse caries assessment methods and multiple OHRQoL instruments, which increases clinical and methodological heterogeneity. A focused synthesis limited to preschool children, ECC and ECOHIS may therefore provide a more clinically interpretable summary of the evidence [10]. The aim of this systematic review was to evaluate whether the presence and/or severity of early childhood caries is associated with poorer oral health-related quality of life in preschool children younger than 6 years of age, as measured by ECOHIS.

## 2. Materials and Methods

### 2.1. Protocol and Reporting Standard

This systematic review was designed in accordance with the Preferred Reporting Items for Systematic Reviews and Meta-Analyses (PRISMA 2020) statement, and the PRISMA 2020 Checklist is included in the Supplementary Materials [11]. The review was structured using the PECO framework for observational studies. The review protocol was not prospectively registered before the study was initiated; however, to enhance methodological transparency, a retrospective registration was completed in the Open Science Framework (OSF) after completion of the review procedures (DOI: 10.17605/OSF.IO/895NV). The registration record is publicly available, and no separate prospectively developed protocol was published. No formal amendments to a prospectively registered protocol were applicable.

The review question was as follows: Among preschool children, is the presence and/or severity of early childhood caries (ECC) associated with poorer oral health-related quality of life measured with ECOHIS?

Eligibility criteria:

- studies including preschool children up to 6 years of age (ECC definition);
- assessed early childhood caries (ECC), caries experience, caries severity, or clinical consequences of untreated caries by means of a clinical dental examination;
- assessed oral health-related quality of life using the Early Childhood Oral Health Impact Scale (ECOHIS) or a validated language or cultural adaptation;
- reported an association between ECC presence and/or severity and ECOHIS scores, or provided sufficient data to determine such an association;
- observational studies, including cross-sectional, case–control, or cohort studies;
- published in English.

The following papers were excluded:

- studies involving school children older than 6 years of age, adolescents, or mixed-age populations without separate preschool data;
- studies using OHRQoL instruments other than ECOHIS;
- studies not evaluating ECC clinically;
- intervention studies, case reports, case series, editorials, letters, conference abstracts, narrative reviews, systematic reviews, and meta-analyses;
- studies with insufficient outcome data

The PECO framework used in this review was defined as follows:

Population (P): preschool children younger than 6 years of age;
Exposure (E): presence and/or severity of early childhood caries;
Comparison (Co): caries-free children or children with lower caries severity;
Outcome (O): oral health-related quality of life measured using ECOHIS.

### 2.2. Information Sources and Search Strategy

A systematic electronic search was conducted in PubMed, Scopus, and Web of Science for studies published within the last 10 years. The search covered the period from 2016 to April 2026 and was last updated on 23 April 2026.

The search included combinations of the following keywords and Medical Subject Headings (MeSH), where applicable: early childhood caries, dental caries, preschool children, oral health-related quality of life, and ECOHIS. Year limits and any additional filters were applied using the database filter tools rather than embedded directly in the search string. The search strategy is presented in the Supplementary Material. Reference lists of included articles were also screened manually to identify additional relevant studies. Grey literature was not searched or included. The review was intentionally restricted to peer-reviewed published studies indexed in the selected bibliographic databases in order to improve methodological consistency and comparability of the included evidence.

### 2.3. Study Selection

All identified records were screened and duplicates were removed. Two reviewers independently screened titles and abstracts for relevance (PPZ and AS). Full texts of potentially eligible studies were then assessed independently according to the predefined inclusion and exclusion criteria. Disagreements were resolved through discussion, and when necessary, by consultation with a third reviewer (ATS).

### 2.4. Data Extraction

Data extraction was performed using a standardized and extraction form developed for this review. Data were extracted independently by two reviewers (PPZ and AS) using a standardized form. The following information was collected from each included study: year of publication, country, study design, sample size, age of participants, caries assessment method, ECC definition or severity measure, ECOHIS outcomes, and the main findings regarding the association between ECC and oral health-related quality of life. Inter-reviewer agreement was assessed using Cohen’s kappa, with agreement at a level of at least 0.80, indicating substantial to almost perfect agreement.

### 2.5. Risk of Bias Assessment

Risk of bias was assessed independently by two reviewers (PPZ and ATS) using the revised JBI critical appraisal tools, according to study design. The revised JBI critical appraisal tool for analytical cross-sectional studies was used for cross-sectional studies, and the revised JBI critical appraisal tool for cohort studies was applied for cohort studies. Any disagreements were resolved by discussion, and, where necessary, by consultation with a third reviewer (DOK). Because the JBI tools do not recommend a single universal numerical cut-off for overall study classification, the final judgment was based on the pattern of item-level responses across domains rather than on a purely mechanical score. Studies with predominantly favorable judgments and no major methodological concerns were classified as low risk of bias; studies with some important concerns in one or more domains, particularly regarding confounding, sampling, or measurement clarity, were classified as moderate risk of bias; and studies with multiple or more serious concerns across key domains were classified as high risk of bias.

### 2.6. Synthesis of the Results

OHRQoL was assessed using the Early Childhood Oral Health Impact Scale (ECOHIS) or its validated language adaptations. In all versions of the instrument, higher ECOHIS scores indicate poorer oral health-related quality of life. A formal meta-analysis was not performed because of substantial methodological heterogeneity across the included studies. The studies differed in caries diagnostic systems and severity classifications (e.g., dmft/dmfs, ICDAS, ICCMS, CAST, and pufa), in the way OHRQoL outcomes were reported (total ECOHIS scores, child and family subscale scores, domain-specific outcomes, dichotomized ECOHIS variables, and adjusted regression estimates), and in their analytical approaches and covariate adjustment strategies. Because these differences limited statistical comparability and made the derivation of a meaningful pooled estimate inappropriate, the findings were synthesized using a structured narrative approach informed by the SWiM framework (Synthesis Without Meta-analysis).

## 3. Results

**Figure 1:**
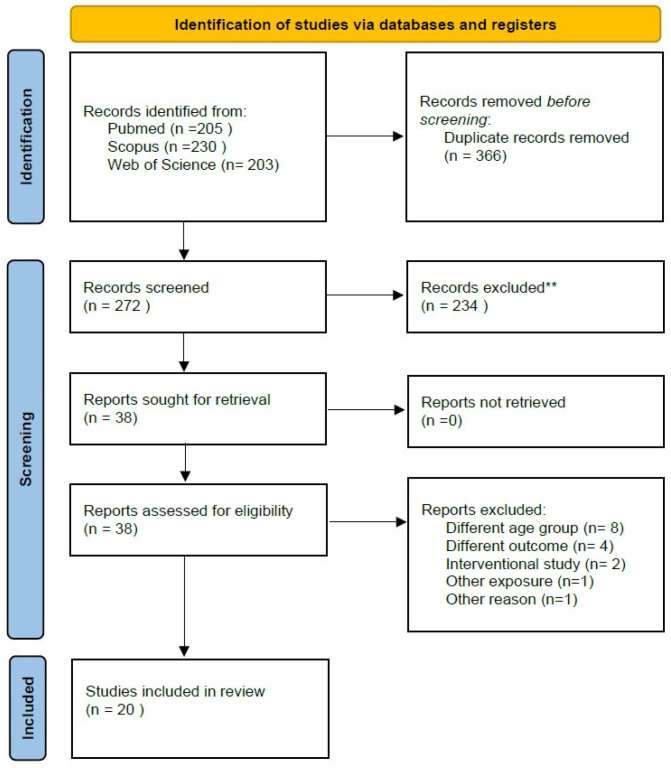
Prisma Flow Diagram. (**: after title or abstract screening).

The literature search identified 638 articles of potential interest, of which 20 were eligible for inclusion in this review [3,4,5,6,7,12,13,14,15,16,17,18,19,20,21,22,23,24,25,26]. The PRISMA flowchart is shown in Figure 1, and the main characteristics of the included studies are listed in Table 1. Most studies used a cross-sectional design, while one study had a cohort design with follow-up. Sample sizes varied considerably, from 151 to 1783 participants, indicating substantial heterogeneity in study scale. The included populations consistently involved preschool-aged children, most commonly between 3 and 5 years of age, although some studies included younger children from infancy or toddlers and, in a few cases, children up to 6 years of age.

Table 2 shows that, despite methodological variation in caries assessment, the included studies consistently demonstrated a negative association between early childhood caries and oral health-related quality of life measured with ECOHIS or its validated language versions. Caries severity was assessed using different diagnostic systems, including dmft, ICDAS, ICDAS-based severity thresholds, CAST, pufa, and related categorizations of untreated disease, but the overall direction of findings was highly consistent across studies. In general, children with caries, and especially those with more severe, extensive, or untreated lesions, had higher total ECOHIS scores and worse child- and family-level impacts than caries-free children. This pattern was observed in cross-sectional studies from different geographic settings and was also supported by longitudinal evidence showing worsening OHRQoL in children who developed incident extensive caries over time (Table 2).

Across the included studies, the presence of ECC or caries experience was consistently associated with poorer OHRQoL in preschool children and their families. Studies using dmft-based comparisons, as well as those based on ICDAS- or CAST-related severity classifications, generally showed higher ECOHIS scores among children with caries than among caries-free children. This pattern was observed across community-based and clinic-based samples and in different language versions of ECOHIS. Representative examples include studies by Rajab et al. [19], Duangthip et al. [17] and Sabel et al. [25], all of which reported worse total or subscale ECOHIS scores among children with caries.

A severity gradient was observed across most studies that compared different levels of caries burden. Children with more extensive disease, severe ECC, higher dmft scores, or dentinal/advanced lesions generally had worse OHRQoL than those with milder disease or no caries. This trend was reported in studies using conventional dmft thresholds as well as more detailed severity systems such as ICDAS and ICCMS. For example, Magdy et al. [7] found worse ECOHIS scores in children with severe ECC than in those with ECC or no caries, while Lara et al. [15] and Díaz et al. [12] reported poorer OHRQoL with increasing lesion severity.

Studies assessing untreated or clinically advanced disease suggested that OHRQoL impairment was greater when caries had progressed to more destructive or symptomatic stages. This was particularly evident in studies using pufa, CAST, or analyses of untreated lesions. In these studies, clinical consequences such as pulpal involvement, pain, or extensive untreated lesions were associated with higher ECOHIS scores than caries experience alone. Alanzi et al. [5] and Sharna et al. [22] found worse quality of life in children with untreated disease consequences, while Pesaressi et al. [21] reported increasing P-ECOHIS scores with higher CAST severity.

Because of the marked heterogeneity in study design, exposure definitions, ECOHIS versions, and reported outcome metrics, no meta-analysis was performed. Instead, the findings were synthesized narratively, and the main quantitative effect estimates reported across studies are summarized in Supplementary Material Table S1 (where applicable).

Risk of bias in the cross-sectional studies was assessed using the revised Joanna Briggs Institute (JBI) Critical Appraisal Checklist for Analytical Cross-Sectional Studies. A total of 19 cross-sectional studies were evaluated with this tool, and the detailed item-by-item assessment is presented in Table 3. Twelve studies were judged as having low risk of bias, 6 as having moderate risk of bias, and 1 as having high risk of bias. Overall, most studies clearly described the study setting and participants, used standard clinical criteria for caries assessment, applied validated versions of ECOHIS, and performed appropriate statistical analyses. The main concerns in studies rated as moderate or high risk of bias were related to insufficient identification or control of confounding factors, unclear reliability of exposure assessment, convenience or clinic-based sampling, and limited reporting of methodological details. Briefly, studies judged as having a moderate risk of bias were typically downgraded because of limited identification or adjustment for confounding factors, clinic-based or convenience sampling, or incomplete methodological detail regarding exposure measurement. The study judged as high risk of bias showed more substantial concerns, particularly related to confounding control, measurement clarity, and overall methodological transparency.

The only cohort study included in the review by Fernandes et al. [14] was assessed as having a moderate risk of bias. Its main strengths were the prospective design, validated OHRQoL instrument, calibrated clinical assessment, and adjusted multivariable analyses. The main concerns related to incomplete follow-up, potential residual confounding, and the use of parent-reported OHRQoL measures.

## 4. Discussion

The present review showed a consistent association between early childhood caries and poorer oral health-related quality of life in preschool children and their families, regardless of the country, study setting, or caries assessment system used. Studies based on dmft categories [16,19,24] reported a clear gradient in which higher caries experience was associated with worse ECOHIS scores. A similar pattern was observed in studies using more detailed clinical systems, including ICDAS, ICCMS, and CAST. Lara et al. [15] found that children with dentinal lesions had markedly worse M-ECOHIS scores than caries-free children, while Díaz et al. [12] showed that extensive lesions were associated with significantly poorer total and domain ECOHIS scores, irrespective of lesion location. These findings suggest that the negative impact of ECC on OHRQoL is robust across different methodological approaches and is not confined to one diagnostic index alone.

An important finding emerging from the included studies is that the burden on OHRQoL was not determined only by the presence of caries, but also by its severity, extension, and clinical consequences. In a report by Magdy et al. [7], children with severe ECC had much higher ECOHIS scores than children with ECC and caries-free children, indicating a marked severity gradient. Alanzi et al. [5] stated that both general caries experience and the severity of untreated disease were associated with worse child- and family-level A-ECOHIS scores, and the pufa index provided additional information beyond dmft alone. A similar pattern was found by Sharna et al. [22], where children with pufa > 0 had substantially worse ECOHIS scores than those without pulpo-periapical consequences of untreated caries, and ECOHIS correlated more strongly with pufa than with defs. These results are clinically important because they indicate that the effect of ECC on daily life is amplified once the disease becomes more advanced, symptomatic, or biologically destructive. In other words, the review suggests that OHRQoL reflects not just whether caries is present, but how far the disease has progressed.

The domains most consistently affected across studies were those related to pain and functional limitation. Frequently reported impacts included tooth pain, difficulty tolerating hot or cold drinks, eating problems, sleep disturbance, and irritability, as shown in the study by Magdy et al. [7]. In the Swedish study [25], caregivers of children with caries reported significantly worse scores across all S-ECOHIS domains than caregivers of caries-free children, while untreated caries was associated with even poorer scores than treated caries. Higher dmft, pufa, and ICDAS scores were also linked to oral pain, difficulties with drinking and eating, sleeping problems, and irritability or frustration, as demonstrated by Kurt et al. [3]. Similarly, in children with S-ECC, both the presence of pain and higher dmft were strongly associated with worse total ECOHIS scores. Taken together, this repeated pattern suggests that, in preschool children, ECC affects quality of life primarily through discomfort, altered eating behavior, sleep disruption, and behavioral distress, rather than through more complex social or self-image domains alone. Socioeconomic and family-related factors appear to modify, but not eliminate, the negative association between caries and OHRQoL. Pereira et al. [18] found that caries experience remained associated with poorer child and family OHRQoL even after considering maternal and socioeconomic characteristics. In Raji et al. [6], higher dmft was associated with poorer child and parent OHRQoL, while household income also contributed to parent-reported burden. Almutairi et al. [26] suggested that broader family functioning, especially roles and problem solving, may influence how oral conditions affect family life. Thus, the evidence in this review supports a model in which ECC is the central clinical driver of poorer OHRQoL, while social and family circumstances shape the magnitude and expression of that burden.

Another point worth emphasizing is that not all studies approached the ECC–OHRQoL relationship in a purely cross-sectional way. Although the main body of evidence was cross-sectional, the longitudinal study by Fernandes et al. [14] adds important support to the interpretation of causally meaningful burden. In that study, the incidence of extensive caries and failure to undergo recommended dental treatment was associated with worsening and severe worsening of B-ECOHIS over a three-year period. This finding strengthens the argument that poorer OHRQoL is not merely an accompanying feature of cross-sectional disease status but may worsen as disease progresses or remains untreated over time. Similarly, Rodrigues et al. [4] expanded the interpretation of the pathway linking ECC and OHRQoL by showing that more severe ECC was associated with dental pain, which in turn was associated with disturbed sleep, and that both ECC and pain negatively affected child and family OHRQoL.

The only cohort study included in this review should also be interpreted in light of follow-up completeness. In the study by Fernandes et al., 151 of 172 children completed the 3-year follow-up, corresponding to a loss of 12.2% of the original sample. The authors reported reasons for attrition, mainly changes in address or telephone number, and compared the baseline characteristics of participants who remained in the study with those lost to follow-up. Nevertheless, incomplete follow-up may still have introduced attrition bias if children lost during follow-up differed systematically from those retained in ways not fully captured by the reported comparisons. For this reason, although the study provides valuable longitudinal support for the association between incident extensive caries and worsening OHRQoL, its findings should be interpreted with some caution.

An important methodological issue in this review is the diagnostic heterogeneity of ECC across the included studies. Although all studies examined the relationship between caries and OHRQoL, they used different systems to define and classify disease burden, including dmft/dmfs, ICDAS, ICCMS, CAST, and pufa. These approaches are not fully interchangeable. Studies based on dmft/dmfs mainly captured cumulative cavitated caries experience and provided relatively broad severity groupings, as seen in studies such as those by Raji et al. [6], Rajab et al. [19], and Duangthip et al. [17]. By contrast, ICDAS- and ICCMS-based studies, including those by Kurt et al. [3], Fernandes et al. [14], and Rodrigues et al. [4], allowed a more refined distinction between enamel, dentinal, moderate, and extensive lesions, thereby capturing severity gradients in greater clinical detail. Although all included studies used ECOHIS or a validated language adaptation, score comparability may still have been influenced by cross-cultural and linguistic differences in caregiver interpretation of symptoms, family burden, and response thresholds. Thus, similar absolute ECOHIS scores across countries should be interpreted cautiously. The sample source may also have affected the observed magnitude of association. Clinic-based studies are more likely to include children with symptomatic, advanced, or treatment-seeking disease, whereas community-based studies may capture a broader spectrum including milder or asymptomatic cases. This difference may partly explain variation in the strength of reported associations across studies.

An interesting complementary longitudinal perspective was provided by Vieira-Andrade et al. [27], although this study was not included in the main synthesis because it examined the reverse temporal direction of the association. Instead of analyzing whether caries worsened OHRQoL, the authors evaluated whether baseline OHRQoL could predict the incidence of untreated dental caries over time. Their findings showed that a higher baseline B-ECOHIS score was associated with a greater risk of developing new untreated carious lesions after two years, together with lower household income and greater baseline caries severity. These results suggest that OHRQoL may not only reflect the current burden of disease, but may also capture broader vulnerability related to future oral health deterioration.

A limitation of this review is that the protocol was not prospectively registered. Although the review was later retrospectively registered in the Open Science Framework, the lack of prior registration may have reduced methodological transparency and may have increased the risk of reporting bias. Publication bias was not formally assessed in this review. Because of the substantial heterogeneity across studies, a funnel plot was not considered appropriate; however, selective publication remains possible, especially given the exclusion of grey literature. In addition, limiting the search to the last 10 years, although intended to focus on the most recent evidence and avoid duplication of data already covered in earlier reviews, may have resulted in the omission of some older relevant studies. The findings of the presented systematic review should be interpreted with caution, as most of the included studies had a cross-sectional design. Therefore, the review supports an association between ECC and poorer OHRQoL, but does not allow confident causal inference regarding the direction or magnitude of this relationship.

The interpretation of the present findings should take into account the methodological limitations identified in the risk of bias assessment. Several studies were based on convenience or clinic-based samples, which may have limited representativeness and generalizability. In addition, socioeconomic factors may have acted as important confounders, while OHRQoL was assessed almost exclusively through caregiver proxy reports, which may not fully reflect the child’s subjective experience. Not all studies adjusted for relevant family- and behavior-related variables, such as parental education, family functioning, oral hygiene routines, or dietary habits. Furthermore, no formal assessment of the overall certainty of evidence, such as GRADE, was performed. Therefore, although the included studies consistently supported an association between greater ECC burden and poorer OHRQoL, the findings should be interpreted as a structured synthesis of predominantly observational evidence rather than as certainty-graded conclusions.

## 5. Conclusions

The findings of this review should be interpreted in light of the methodological heterogeneity. The included studies differed in sample size, recruitment setting, caries thresholds, and severity classifications, and some relied on broad dmft grouping while others used other approaches. Nevertheless, the direction of findings remained remarkably stable. This consistency is important because it suggests that the association between ECC and worse OHRQoL is not an artifact of one particular measurement strategy. At the same time, the review highlights the value of more refined diagnostic approaches. This may explain why some studies were able to demonstrate not only that ECC impairs OHRQoL, but also that advanced, extensive, or symptomatic disease produces disproportionately greater burden. The available evidence consistently indicates that ECC is associated with poorer OHRQoL in preschool children and their families, particularly when the disease is more severe or remains untreated. However, because most included studies were cross-sectional, these findings should be interpreted as associative rather than causal.

## Figures and Tables

**Table 1:** Population and study characteristics of the included studies.

First Author, Year	Country	Study Design	Setting	Sample Size	Age of Participants	Study Characteristics
Alanzi, 2026 ([5])	Kuwait	National cross-sectional study	Randomly selected kindergarten schools across all six governorates in Kuwait	1783	4–5 years	Healthy, cooperative Kuwaiti kindergarten children; non-cooperative children and those with systemic disease were excluded
Magdy, 2026 ([7])	Egypt	Cross-sectional observational study	Pediatric Dentistry Outpatient Clinic, Ain Shams University; The British University in Egypt	260	3–5 years	Preschool children attending outpatient pediatric dental clinic; ECC and caries-free children included for comparison
Raji, 2026 ([6])	Iran	Cross-sectional study	Persian birth cohort-Isfahan	347	2–6 years	Preschool children from a range of socioeconomic backgrounds; subset of birth cohort; severe sleep disorders, dental trauma and clinically significant malocclusion excluded
Díaz, 2025 ([12])	Colombia	Cross-sectional analytical study	Representative sample of caregiver–child dyads; preschool population in Colombia	643	0–5 years	Preschool caregiver–child dyads; children assessed for caries stage and lesion location
Kurt, 2025 ([3])	Turkey	Cross-sectional study	Pediatric Dentistry Department, Recep Tayyip Erdoğan University Faculty of Dentistry	300	3–6 years	Children aged 3–6 years and their parents/caregivers; uncooperative children, children with systemic diseases, enamel anomalies were excluded
Rodrigues, 2025 ([4])	Brazil	Cross-sectional study	Public and private schools in the urban area; two-stage random sampling	575	3–5 years	Children enrolled in public and private urban schools; excluded were children who did not cooperate with examination, refused participation, were taking medications that could affect sleep, or had cognitive and/or motor disabilities
Sabel, 2024 ([25])	Sweden	Cross-sectional study	Three dental clinics in the Public Dental Service of Region Västra Götaland and Region Halland, Sweden	274	2–5 years	Preschool children and their caregivers; 117 children had caries (dmft > 0) and 157 were caries-free (dmft = 0)
Alanzi, 2023 ([13])	Kuwait	Cross-sectional study	Preselected public schools/kindergartens from one Governorate in Kuwait	334	4–5 years	Kindergarten level-1 (KG1) and level-2 (KG2) children; demographic information was collected from caregivers
Almutairi, 2023 ([26])	United Kingdom	Cross-sectional study	East London Oral Health Inequalities (ELOHI) study; community-based survey in Outer North East London	740	3–4 years	Parent–child dyads from the general non-institutionalized population; children underwent clinical examination and were classified as dmft = 0 or dmft > 0
Fernandes, 2023 ([14])	Brazil	Cohort study	Diamantina, Brazil; 3-year follow-up	151	1–3 years	Preschool children and their mothers; mothers completed B-ECOHIS and socio-environmental questionnaire; children examined for caries and other oral conditions
Lara, 2022 ([15])	Mexico	Cross-sectional study	Preschool children in Mexico	409	3–5 years	Preschool children; caries severity assessed at two ICDAS thresholds; caregivers completed the Mexican ECOHIS (M-ECOHIS)
Pakkhesal, 2021 ([16])	Iran	Descriptive-analytical cross-sectional study	Licensed kindergartens in Gorgan, selected using proportional allocation sampling	350	3–6 years	Parents completed the Persian ECOHIS; children with systemic disease, specific medication use, and incomplete parent questionnaires were excluded
Rajab, 2020 ([19])	Jordan	Cross-sectional study	Preschools in Amman, Jordan	1557	4–5 years	Parents/caregivers completed ECOHIS and sociodemographic questionnaire; malocclusion traits and traumatic dental injuries were also assessed
Duangthip, 2020 ([17])	Hong-Kong, China	Cross-sectional study	Hong Kong preschools/kindergartens; children were examined in their classrooms	336	mean age 4.7 (0.3) years	Preschool children whose parents or primary caregivers completed the Chinese ECOHIS and a dental health questionnaire; children with major systemic illnesses or refusal of examination were excluded
Pereira, 2020 ([18])	Brazil	Cross-sectional study	Public Primary Care Services in Porto Alegre, Southern Brazil	163	3–4 years	reschool children and their mothers; mothers completed the Brazilian ECOHIS and questionnaires on sociodemographic and maternal behavior characteristics; caries severity was assessed using ICDAS
Lai, 2019 ([20])	Hong Kong, China	Cross-sectional study	Faculty of Dentistry, The University of Hong Kong	315	Mean age 4.7 ± 0.8 years	Preschool children with severe early childhood caries; caries and plaque status were evaluated, and pain status was also recorded
Pesaressi, 2019 ([21])	Peru	Analytical observational study	Government preschools in low socioeconomic status districts in Lima, Peru	213/308	Mean age 3.04 years	Longitudinal study of three-year-old preschool children from low-SES districts
Sharna, 2019 ([22])	India	Cross-sectional study	Outpatient clinic of the Department of Pediatric and Preventive Dentistry	238	6–72 months	Children with ECC and; medically compromised children were excluded
Antunes, 2018 ([23])	Brazil	Cross-sectional study	Public day care centers in a city in the Southeast region of Brazil	446	2–6 years	Preschool children attending public day care centers; children were classified according to ECC severity
Chaffee, 2017 ([24])	Brazil	Cross-sectional study	Southern Brazil	456	Mean age 38 months	Preschool children and their mothers; children were grouped by caries experience (dmft = 0, 1–4, and ≥5), and analyses were stratified by socioeconomic indicators including maternal education, social class, and household income

**Table 2:** Caries assessment, OHRQoL measurement and main findings of the included studies.

First Author, Year	Caries Assessment Method	ECC Severity Measure	OHRQoL Instrument	Main Outcome(s) Reported	Main Findings
Alanzi, 2026 ([5])	dmft/dmfs + merged ICDAS + pufa	ECC prevalence 88.6%; mean dmft 6.45 ± 4.5, dmfs 13.0 ± 14.1; ICDAS 5–6: 63.4%; PUFA > 0: 9.3%	A-ECOHIS	Child- and family-level OHRQoL	Caries experience was associated with higher child and family A-ECOHIS scores (*p* < 0.001); pufa was associated with poorer child-level, but not family-level, OHRQoL
Magdy, 2026 ([7])	dmft (WHO criteria	Caries-free/ECC/S-ECC; 75.8% had ECC, of whom 31% had ECC and 69% had S-ECC	A-ECOHIS	Association of ECC presence/severity and dmft with OHRQoL	Mean ECOHIS 19.52 ± 12.46; S-ECC showed the worst OHRQoL (25.87 ± 10.05), followed by ECC (20.35 ± 8.96), versus caries-free children (4.94 ± 6.08); worse OHRQoL was associated with ECC presence and higher dmft scores
Raji, 2026 ([6])	dmft	Caries severity analyzed as dmft score	Persian ECOHIS	Association of dmft with child and parent/family OHRQoL	Higher dmft was associated with lower child and parent OHRQoL (*p* < 0.001); each 1-unit increase in dmft increased the odds of low child OHRQoL (OR 1.23), low parent OHRQoL (OR 1.32), and lower parental satisfaction (OR 1.35)
Díaz, 2025 ([12])	ICDAS + ICCMS	Sound/Initial (A)/Moderate (B)/Extensive (C), with lesion location by anterior/posterior/both; caries prevalence 72.6%	Colombian Spanish ECOHIS	Association of caries stage and location with OHRQoL	Extensive lesions were associated with worse total and domain ECOHIS scores (*p* < 0.001); moderate lesions affected OHRQoL only when present in both anterior and posterior teeth (RR 1.54; *p* = 0.045); TDI was also independently associated with poorer OHRQoL (RR 1.63; *p* < 0.001)
Kurt, 2025 ([3])	dmft + pufa + ICDAS II	Caries severity/extension assessed with dmft, pufa and ICDAS II; mean dmft 4.82, mean pufa 0.41	ECOHIS	Association of caries severity/extension with OHRQoL	Higher dmft, pufa and ICDAS scores were associated with worse OHRQoL; pufa ≥ 1 predicted ECOHIS ≥ 1 (adjusted OR 1.929, *p* = 0.039), and dmft also predicted higher ECOHIS scores (adjusted OR 6.597, *p* = 0.048)
Rodrigues, 2025 ([4])	ICDAS-II	More severe ECC stages analyzed in SEM model	ECOHIS	Association of ECC, dental pain and sleep problems with child/family OHRQoL	More severe ECC was associated with dental pain (β = 0.712), which was associated with disturbed sleep (β = 0.723); ECC and dental pain both worsened child OHRQoL (β = 0.208; β = 0.750) and family OHRQoL (β = 0.347; β = 0.612), all *p* < 0.001
Sabel, 2024 ([25])	dmft	Caries group (dmft > 0) vs. non-caries group (dmft = 0); additionally treated caries (d = 0) vs. untreated caries (d > 0); mean dmft 2.0 overall and 4.7 in caries group	S-ECOHIS	Comparison of child and family OHRQoL by caries status	Children with caries had worse OHRQoL than caries-free children (total S-ECOHIS 5.97 vs. 0.77; *p* < 0.001); untreated caries showed worse OHRQoL than treated caries (6.60 vs. 2.50; *p* < 0.001)
Alanzi, 2023 ([13])	dmft/dmfs + pufa + degrees of untreated caries (dt)	Caries prevalence 78.9% in KG1 and 67.4% in KG2; 29.6% caries-free, 37.8% non-severe, 32.6% severe; mean dmft 4.32 ± 4.4, dmfs 9.46 ± 11.1; 19.2% had pufa > 0	A-ECOHIS	Association of caries experience and untreated caries severity with OHRQoL	Child and family ECOHIS scores were higher for children with dmft ≥ 1 and pufa ≥ 1 (both *p* < 0.001); child impact scores increased with increasing untreated caries severity (dt; *p* = 0.004)
Almutairi, 2023 ([26])	dmft	Caries experience analyzed as dmft = 0 vs. dmft > 0; 24.8% had caries experience	ECOHIS	Association of family functioning and caries experience with child/family OHRQoL	Children with dmft > 0 had worse ECOHIS scores; in adjusted models, caries experience was associated with higher total ECOHIS (RR 3.72), CIS (RR 2.77), and FIS (RR 7.44)
Fernandes, 2023 ([14])	ICDAS-II	Extensive dental caries defined as ICDAS 5–6; baseline extensive caries prevalence 27.8%	B-ECOHIS	Worsening and severe worsening of OHRQoL over time	Mean B-ECOHIS increased from 3.1 to 4.1; worsening occurred in 37.7% and severe worsening in 25.8%; incident extensive caries predicted worsening (RR 1.91) and severe worsening (RR 2.06)
Lara, 2022 ([15])	ICDAS-II + dmft	Caries-free/enamel lesions/dentinal lesions; caries prevalence 82.2% overall and 45.0% for dentinal lesions	M-ECOHIS	Association of ECC severity with child/family OHRQoL	M-ECOHIS scores increased significantly with caries severity (χ^2^(4) = 175.85, *p* < 0.01); high scores were concentrated in children with dentinal lesions, while most caries-free children had low M-ECOHIS scores
Pakkhesal, 2021 ([16])	dmft	Caries-free (dmft = 0), 1 ≤ dmft ≤ 5, and dmft ≥ 6; mean dmft 3.94 ± 4.17	ECOHIS	Association of caries severity with child and family OHRQoL	Mean total ECOHIS was 11.88 ± 6.91; scores increased with caries severity (9.29, 12.38, 13.97 respectively); family impact increased significantly across severity groups (*p* < 0.001), and higher dmft was associated with worse OHRQoL
Rajab, 2020 ([19])	dmft	Caries-free (dmft = 0), dmft 1–4, and dmft > 4; ECC prevalence 72.5% at age 4 and 77.2% at age 5	Arabic ECOHIS	Association of ECC presence/severity with child and family OHRQoL	Children with ECC had higher scores for all ECOHIS items and overall ECOHIS than caries-free children; scores increased with severity, and dental caries was the only significant predictor of worse OHRQoL (OR 4.0, 95% CI 3.179–5.972, *p* < 0.001)
Duangthip, 2020 ([17])	dmft	Caries prevalence analyzed as dmft > 0 and by dmft score; prevalence 36.9%, mean dmft 1.7 ± 3.2	Chinese ECOHIS	Association of caries experience with poor OHRQoL (ECOHIS > 0)	ECOHIS impact was reported in 70.2% of children; mean ECOHIS 5.8 ± 6.2; higher dmft increased the odds of poorer OHRQoL (OR 1.20, 95% CI 1.07–1.35, *p* = 0.002)
Pereira, 2020 ([18])	ICDAS	ECC defined as ICDAS ≥ 1; ECC prevalence 91.4%	B-ECOHIS	Association of ECC and maternal/socioeconomic factors with OHRQoL	Children with caries had higher function and parental anxiety domain scores; child caries experience was associated with worse child/family OHRQoL (RR 2.21; 95% CI 1.43–3.41)
Lai, 2019 ([20])	dmft + VPI + pain	All children had S-ECC; mean dmft 10.2 ± 4.5; 98.7% had decayed teeth, 61.4% had VPI > 90%, and 28.9% had pain	ECOHIS	Association of S-ECC severity and pain with child/family OHRQoL	Higher dmft and pain were associated with worse OHRQoL; in multiple regression, total ECOHIS was associated with dmft (β = 0.490, *p* < 0.001) and pain (β = 4.698, *p* < 0.001)
Pesaressi, 2019 ([21])	CAST	Caries prevalence 64.3% (CAST 4–7); severity analyzed by MaxCAST codes 3, 5, and 6	P-ECOHIS	Association of caries severity with child and family OHRQoL	P-ECOHIS scores increased with caries severity; children with higher MaxCAST scores had significantly worse family and total impact scores than those with lower severity (family impact *p* = 0.003; total impact *p* = 0.001)
Sharna, 2019 ([22])	defs + pufa	All children had ECC; severity analyzed as pufa = 0 vs. pufa > 0 and by continuous pufa/defs scores	ECOHIS	Comparison of OHRQoL by pulpo-periapical consequences of untreated caries	Mean ECOHIS was 14.12 ± 6.72; children with pufa > 0 had worse OHRQoL than those with pufa = 0 (16.14 vs. 9.07; *p* < 0.001); ECOHIS correlated more strongly with pufa (ρ = 0.431) than with defs (ρ = 0.288)
Antunes, 2018 ([23])	WHO 2013 criteria	Caries-free/low-severity ECC/high-severity ECC; prevalence 33.7%; low severity OR 1.71 and high severity OR 5.78 for negative OHRQoL impact	B-ECOHIS	Association of ECC severity with OHRQoL	ECOHIS scores were significantly associated with ECC severity; higher-severity ECC showed substantially greater negative impact on OHRQoL, and clinical plus socio-dental indicators should be interpreted together
Chaffee, 2017 ([24])	dmft	Caries-free (dmft = 0), dmft 1–4, and dmft ≥ 5; caries prevalence 39.7%, mean dmft 1.54 ± 2.77	Brazilian ECOHIS	Association of caries severity and socioeconomic status with child/family OHRQoL	Mean ECOHIS was 2.0 ± 3.5; increasing caries severity worsened OHRQoL, and children with dmft ≥ 5 had 3.0-fold higher ECOHIS scores than caries-free children (95% CI 2.0–4.4); this pattern persisted across SES strata

Legends: ECOHIS—Early Childhood Oral Health Impact Scale. A-ECOHIS—Arabic version of the Early Childhood Oral Health Impact Scale. S-ECOHIS—Swedish version of the Early Childhood Oral Health Impact Scale. B-ECOHIS—Brazilian version of the Early Childhood Oral Health Impact Scale. M-ECOHIS—Mexican version of the Early Childhood Oral Health Impact Scale. P-ECOHIS—Peruvian version of the Early Childhood Oral Health Impact Scale.

**Table 3:** Risk of bias assessment of cross-sectional studies using the revised JBI Critical Appraisal Checklist.

First Author, Year	Q1	Q2	Q3	Q4	Q5	Q6	Q7	Q8	Overall Appraisal
Alanzi, 2026 ([5])	Y	Y	Y	Y	Y	Y	Y	Y	Low risk of bias
Magdy, 2026 ([7])	Y	Y	Y	Y	Y	Y	Y	Y	Low risk of bias
Raji, 2026 ([6])	Y	Y	Y	Y	Y	Y	Y	Y	Low risk of bias
Díaz, 2025 ([12])	Y	Y	Y	Y	Y	Y	Y	Y	Low risk of bias
Kurt, 2025 ([3])	Y	Y	Y	Y	Y	Y	Y	Y	Low risk of bias
Rodrigues, 2025 ([4])	Y	Y	Y	Y	Y	Y	Y	Y	Low risk of bias
Sabel, 2024 ([25])	Y	Y	Y	Y	U	N	Y	Y	Moderate risk of bias
Alanzi, 2023 ([13])	Y	Y	Y	Y	Y	Y	Y	Y	Low risk of bias
Almutairi, 2023 ([26])	Y	Y	Y	Y	Y	Y	Y	Y	Low risk of bias
Lara, 2022 ([15])	Y	Y	Y	Y	Y	Y	Y	Y	Low risk of bias
Pakkhesal, 2021 ([16])	Y	Y	Y	Y	U	N	Y	Y	Moderate risk of bias
Rajab, 2020 ([19])	Y	Y	Y	Y	Y	Y	Y	Y	Low risk of bias
Duangthip, 2020 ([17])	Y	Y	U	Y	Y	Y	Y	Y	Moderate risk of bias
Pereira, 2020 ([18])	Y	Y	Y	Y	Y	Y	Y	Y	Low risk of bias
Lai, 2019 ([20])	Y	Y	Y	Y	Y	Y	Y	Y	Low risk of bias
Pesaressi, 2019 ([21])	Y	Y	Y	Y	N	N	Y	Y	Moderate risk of bias
Sharna, 2019 ([22])	Y	Y	U	Y	N	N	U	Y	High risk of bias
Antunes, 2018 ([23])	Y	Y	Y	Y	U	N	Y	Y	Moderate risk of bias
Chaffee, 2017 ([24])	U	Y	Y	Y	U	Y	Y	Y	Moderate risk of bias

Abbreviations: Y, yes; N, no; U, unclear. Q1. Were the criteria for inclusion in the sample clearly defined? Q2. Were the study subjects and the setting described in detail? Q3. Was the exposure measured in a valid and reliable way? Q4. Were objective, standard criteria used for measurement of the condition? Q5. Were confounding factors identified? Q6. Were strategies to deal with confounding factors stated? Q7. Were the outcomes measured in a valid and reliable way? Q8. Was appropriate statistical analysis used?

## Data Availability

No new data were created or analyzed in this study. Data sharing is not applicable to this article.

## Supplementary Materials

The following supporting information can be downloaded at: https://www.mdpi.com/article/10.3390/jcm15114314/s1. PRISMA 2020 Checklist [11]; Supplementary Material S1. Search strategy for databases; Supplementary Table S1. Reported quantitative effect estimates across included studies.

## Abbreviations

The following abbreviations are used in this manuscript:

OHRQoL	Oral health-related quality of life
ECOHIS	The Early Childhood Oral Health Impact Scale
ECC	Early childhood caries
