# Supplementary material for: Early Childhood Caries and Oral Health-Related Quality of Life in Preschool Children: A Systematic Review
Source: J Clin Med. 2026 Jun 2;15(11):4314. doi: 10.3390/jcm15114314 (PMC13257429; doi:10.3390/jcm15114314)
Supplement: Supplementary file 1 [file jcm-15-04314-s001.zip › Supplementary Table S1. Reported quantitative effect estimates across included studies.pdf]

Supplementary Table S2. Reported quantitative effect estimates across included studies.

Study	Comparison	Key reported estimate(s)
Raji, 2026	Per 1-unit increase in dmft	Low child OHRQoL: OR = 1.23 (95% CI: 1.16–1.31); low parent OHRQoL: OR = 1.32 (95% CI: 1.23–1.41); lower parental satisfaction: OR = 1.35 (95% CI: 1.26–1.45)
Díaz, 2025	Moderate lesions in both anterior and posterior teeth; traumatic dental injury	Moderate lesions in both arches: RR = 1.54 (p = 0.045); traumatic dental injury: RR = 1.63 (p < 0.001)
Kurt, 2025	pufa ≥ 1; higher dmft	ECOHIS ≥ 1: adjusted OR = 1.929 (p = 0.039); higher ECOHIS with dmft: adjusted OR = 6.597 (p = 0.048)
Rodrigues, 2025	ECC severity, dental pain, sleep problems	ECC→pain: β = 0.712; pain→sleep problems: β = 0.723; ECC→child OHRQoL: β = 0.208; pain→child OHRQoL: β = 0.750; ECC→family OHRQoL: β = 0.347; pain→family OHRQoL: β = 0.612 (all p < 0.001)
Sabel, 2024	Caries vs no caries; untreated vs treated caries	Total S-ECOHIS: 5.97 ± 6.16 (caries) vs 0.77 ± 1.38 (no caries), p < 0.001; untreated vs treated caries: 6.60 ± 6.39 vs 2.50 ± 3.02, p < 0.001
Almutairi, 2023	dmft > 0 vs dmft = 0	Total ECOHIS: RR = 3.72 (95% CI: 2.20–6.32); CIS: RR = 2.77 (95% CI: 1.60–4.77); FIS: RR = 7.44 (95% CI: 4.10–13.50)
Fernandes, 2023	Incident extensive caries; no dental treatment	Worsening OHRQoL: RR = 1.91 (95% CI: 1.26–2.91); severe worsening: RR = 2.06 (95% CI: 1.05–4.07); no dental treatment and worsening: RR = 2.49 (95% CI: 1.62–3.81); severe worsening: RR = 3.68 (95% CI: 1.96–6.89)
Pakkhesal, 2021	dmft = 0 vs 1–5 vs ≥6	Total ECOHIS: 9.29 ± 5.04 vs 12.38 ± 8.25 vs 13.97 ± 8.89; family impact: 0.87 ± 1.70 vs 2.72 ± 3.25 vs 4.01 ± 3.57 (p < 0.001)
Rajab, 2020	ECC severity categories vs caries-free	Dental caries was the only significant predictor of worse OHRQoL: OR = 4.0 (95% CI: 3.179–5.972, p < 0.001)
Duangthip, 2020	Per increase in dmft	Poor OHRQoL: OR = 1.20 (95% CI: 1.07–1.35, p = 0.002)
Pereira, 2020	ECC present vs absent	Worse child/family OHRQoL: RR = 2.21 (95% CI: 1.43–3.41)
Lai, 2019	Higher dmft; pain	Total ECOHIS: dmft β = 0.490 (p < 0.001); pain β = 4.698 (p < 0.001)
Pesaressi, 2019	Higher CAST severity (MaxCAST 5/6 vs 3)	Higher MaxCAST scores were associated with worse family impact (p = 0.003) and total impact (p = 0.001)
Sharna, 2019	pufa > 0 vs pufa = 0	Total ECOHIS: 16.14 ± 6.27 vs 9.07 ± 4.94 (p < 0.001); correlation with pufa: ρ = 0.431 (p < 0.001); correlation with defs: ρ = 0.288 (p < 0.001)
Antunes, 2018	Low- and high-severity ECC vs caries-free	Low-severity ECC: OR = 1.71 (95% CI: 1.01–2.91, p = 0.03); high-severity ECC: OR = 5.78 (95% CI: 1.65–24.22, p = 0.001)
Chaffee, 2017	dmft ≥ 5 vs dmft = 0	Children with dmft ≥ 5 had 3.0-fold higher ECOHIS scores than caries-free children (95% CI: 2.0–4.4)
