# Supplementary material for: Early Childhood Caries and Oral Health-Related Quality of Life in Preschool Children: A Systematic Review
Source: J Clin Med. 2026 Jun 2;15(11):4314. doi: 10.3390/jcm15114314 (PMC13257429; doi:10.3390/jcm15114314)
Supplement: Supplementary file 1 [file jcm-15-04314-s001.zip › Supplementary Material 1. Search strategy for databases.pdf]

Search strategy for databases

1. **PubMed (PubMed interface)**

(("Dental Caries"[Mesh] OR "early childhood caries"[Title/Abstract] OR ECC[Title/Abstract] OR "dental caries"[Title/Abstract] OR caries[Title/Abstract]) AND ("Child, Preschool"[Mesh] OR preschool*[Title/Abstract] OR "preschool child*"[Title/Abstract] OR toddler*[Title/Abstract] OR "early childhood"[Title/Abstract]) AND ("Early Childhood Oral Health Impact Scale"[Title/Abstract] OR ECOHIS[Title/Abstract] OR "oral health-related quality of life"[Title/Abstract] OR OHRQoL[Title/Abstract]))

2. Scopus (Scopus interface)

TITLE-ABS-KEY (("early childhood caries" OR ECC OR "dental caries" OR caries) AND (preschool* OR "preschool child*" OR toddler* OR "early childhood") AND ("Early Childhood Oral Health Impact Scale" OR ECOHIS OR "oral health-related quality of life" OR OHRQoL))

3. Web of Science Core Collection (Web of Science interface)

TS=(("early childhood caries" OR ECC OR "dental caries" OR caries) AND (preschool* OR "preschool child*" OR toddler* OR "early childhood") AND ("Early Childhood Oral Health Impact Scale" OR ECOHIS OR "oral health-related quality of life" OR OHRQoL))
